# Glutathione Is a Key Player in Metal-Induced Oxidative Stress Defenses

**DOI:** 10.3390/ijms13033145

**Published:** 2012-03-07

**Authors:** Marijke Jozefczak, Tony Remans, Jaco Vangronsveld, Ann Cuypers

**Affiliations:** Centre for Environmental Sciences, Hasselt University, Agoralaan Building D, 3590 Diepenbeek, Belgium; E-Mails: marijke.jozefczak@uhasselt.be (M.J.); tony.remans@uhasselt.be (T.R.); jaco.vangronsveld@uhasselt.be (J.V.)

**Keywords:** metals, cellular redox state, glutathione, chelation

## Abstract

Since the industrial revolution, the production, and consequently the emission of metals, has increased exponentially, overwhelming the natural cycles of metals in many ecosystems. Metals display a diverse array of physico-chemical properties such as essential *versus* non-essential and redox-active *versus* non-redox-active. In general, all metals can lead to toxicity and oxidative stress when taken up in excessive amounts, imposing a serious threat to the environment and human health. In order to cope with different kinds of metals, plants possess defense strategies in which glutathione (GSH; γ-glu-cys-gly) plays a central role as chelating agent, antioxidant and signaling component. Therefore, this review highlights the role of GSH in: (1) metal homeostasis; (2) antioxidative defense; and (3) signal transduction under metal stress. The diverse functions of GSH originate from the sulfhydryl group in cysteine, enabling GSH to chelate metals and participate in redox cycling.

## 1. Introduction

Metals are natural components of the earth’s crust; low background concentrations can be detected in soils, sediments, waters and even in organisms. Due to their versatile applicability, metals are widely used in, amongst others, electronic components, building materials, motor fuels and fertilizers. Since the industrial revolution in 1815, advanced industrial and agricultural activities have exponentially increased the production and consequently the emission of metals. Unlike many organic pollutants, which potentially degrade to carbon dioxide (CO_2_) and water (H_2_O), metals are not biodegradable and persist in the environment. The cumulative industrial release into our environment has been massive and has overwhelmed the natural cycles of metals in many ecosystems [1–3]. Quantification of material extraction for the global economy forecasts a significant growth of resource extraction. From all resources, the extraction of metal ores has proportionally increased the most since 1980. An even larger increase is predicted for the near future, indicating the continued importance of metals for industrial development, with the consequence of increased emissions into the environment [4,5].

Essential metals [cobalt (Co), copper (Cu), iron (Fe), manganese (Mn), molybdenum (Mo), nickel (Ni) and zinc (Zn)] serve as critical micronutrients for normal development and growth of organisms. Since these elements can become toxic at higher levels, plants have developed a strict regulation to absorb, translocate and store them within physiological ranges. Despite the selectivity of transport systems, non-essential metals and metalloids like arsenic (As), cadmium (Cd), chromium (Cr), lead (Pb) and mercury (Hg) also make use of these uptake mechanisms [6]. The main environmental threats are associated with these non-essential elements because plants, as primary producers, form an important entry pathway for potentially toxic substances into the food chain [1,7]. Regardless of this knowledge, metal emissions are still continuing, particularly in less developed countries [8–10].

In order to cope with different kinds of metals, plants possess defense strategies related to the cellular free metal content on one hand (e.g., metal exclusion, cell wall binding, chelation and sequestration [11]) and the regulation of cellular responses on the other hand (e.g., repair of stress-damaged proteins, antioxidative defense [11]). To limit free metal concentrations, the synthesis of specific chelators and subsequent sequestration of metal complexes are of major importance. Glutathione (GSH) is a key component in such metal scavenging due to the high affinity of metals to its thiol (-SH) group and as a precursor of phytochelatins (PCs). Besides metal homeostasis, plants posses a well-equipped antioxidative defense system to manage the metal-imposed oxidative challenge [12–14]. The cysteine residue on GSH renders it an important antioxidant that, in addition to its primary antioxidant capacities, acts as a substrate for the regeneration of other essential antioxidants [13,15,16]. In this way, GSH performs in both metal homeostasis and the antioxidative defense, which influence the levels of free reduced GSH and its cellular redox state [*i.e.*, oxidized glutathione disulfide (GSSG) *versus* reduced GSH]. Furthermore, the GSSG/GSH redox balance transmits specific information in order to fine tune cellular signaling pathways and responses under environmental stress conditions [17,18]. The regulation of the different roles of GSH and the precise mechanisms by which it acts as a signal transducer under metal-induced oxidative stress is currently under intense investigation and will provide essential information to understand the cellular responses to metal toxicity. This review highlights on GSH and its involvement in: (1) metal homeostasis; (2) antioxidative defense; and (3) signaling under metal stress.

## 2. Glutathione Biosynthetic Pathway and Its Regulation

**Figure 1:**
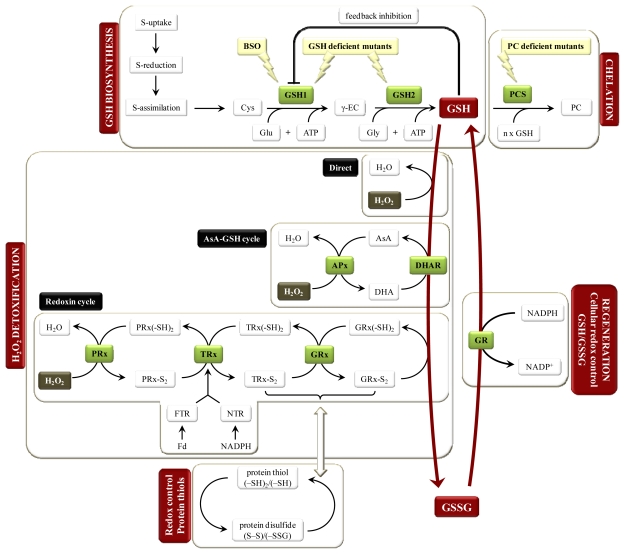
Overview of GSH biosynthesis and its involvement in chelation and redox control. Glutathione is made of three amino acids: γ-glutamate (Glu), cysteine (Cys) and glycine (Gly) by γ-glutamylcysteine synthetase (GSH1) and GSH synthetase (GSH2) and depends on sulfur (S) availability. Multiple GSH molecules are polymerized by phytochelatin synthase (PCS) to form phytochelatins (PCs). Several antioxidative defense pathways are interconnected with GSH in order to remove excess hydrogen peroxide (H_2_O_2_). The first defense pathway represents direct non-enzymatic GSH oxidation. Secondly, the ascorbate(AsA)-GSH cycle is displayed in which AsA and GSH are successively oxidized and reduced to allow AsA peroxidase (APx) to neutralize H_2_O_2_. Thirdly, the two major thiol-redox enzymes glutaredoxin (GRx) and thioredoxin (TRx) are presented that complement the GSH system in redox signaling ether by recycling peroxiredoxin (PRx) that neutralizes H_2_O_2_ or through redox control of protein thiols. In order to investigate the role of GSH in metal stressed plants, inhibitors of GSH synthesis [*i.e.*, buthionine sulfoximine (BSO)] or deficient mutants are used. Abbreviations: dehydroascorbate (DHA), dehydroascorbate reductase (DHAR), ferredoxin (Fd), ferredoxin-dependent thioredoxin reductase (FTR), γ-glutamylcysteine (γ-EC), NADPH-dependent thioredoxin reductase (NTR).

Glutathione (γ-glutamyl-cysteinyl-glycine) is a widely distributed tripeptide found at millimolar concentrations (0.5–10 mM) in plant cells [19]. It is synthesized from three amino acids in two ATP-dependent steps, beginning with the formation of a peptide bond between γ-glutamate and cysteine by γ-glutamylcysteine synthetase (GSH1) and the subsequent addition of glycine, catalyzed by glutathione synthetase (GSH2) (Figure 1: GSH biosynthesis) [20]. Genome sequencing of *Arabidopsis* indicated that both enzymes, GSH1 and GSH2, are encoded by single genes. Mutational knockouts in one of both genes have lethal phenotypes, pointing towards a single pathway for GSH synthesis in plants. Exogenous administration of GSH *in vitro* could partially rescue the development of mutant embryos, demonstrating that endogenous GSH is essential for seed maturation and also during germination [21,22]. Therefore, in order to investigate the importance of GSH under metal stress, GSH-deficient conditions in mutants due to decreased GSH1 activity are preferred instead of knockout plants [23]. Also the GSH1 inhibitor buthionine sulfoximine **(**BSO) is often used to alter the pool of cellular GSH. A study in Cu-exposed *Silene cucubalus* demonstrated increased lipid peroxidation and a more oxidized GSSG/GSH ratio after Cu treatment (20 μM). Depletion of GSH by BSO-pretreatment significantly increased the oxidative damage by Cu [24]. Recently, Wójcik and Tukiendorf (2011) exposed *Arabidopsis* to Cd, with or without addition of BSO, to investigate GSH adaptation to Cd stress (50 and 100 μM). This experiment showed that treatment with BSO increased Cd toxicity and both GSH content and PC accumulation were more than 96% reduced [25]. A study investigating the compartmentalization of GSH biosynthesis shows that GSH1 is restricted to plastids, whereas GSH2 is largely localized in the cytosol and less than 10% will be active in plastids [26]. This observation is consistent with earlier conclusions that export of reduced sulfur from the plastids is mainly in the form of γ-glutamylcysteine (γ-EC), a precursor of GSH [27].

The most important factors affecting GSH synthesis rate are sulfur availability and GSH1 activity. Metal toxicity increases both factors in order to meet the elevated GSH demand in cells to ensure detoxification and survival. Sulfur is taken up by plants from the soil as sulfate. After reduction, it is assimilated into bio-organic compounds, with cysteine being the first product (Figure 1: GSH biosynthesis). Generally, this pathway is regulated by demand for reduced sulfur. Since GSH is an important storage form of reduced sulfur in cells, high demands for GSH due to metal stress stimulate sulfate uptake, reduction and assimilation in order to meet the needs of cysteine for GSH and PC biosynthesis [28,29]. This is consistent with studies in maize roots exposed to Cd, Cu and Zn that demonstrate an increased expression of sulfate transporters (e.g., ST1) accompanied with an elevated sulfate uptake [30–32]. For the reduction of sulfate under Cd stress, increased transcripts of sulfate reductases (e.g., ATPS, APSR) could be linked with alterations in GSH1 [30] and specific isoforms of serine acetyltransferase (SAT) and *O*-acetylserine(thiol)lyase (OASTL), the first and last enzyme of cysteine synthesis, respectively. These findings further support the notion for a coordinate transcriptional regulation of sulfur assimilation and GSH synthesis genes as part of the cellular response to Cd exposure [33,34].

The rate-limiting step of GSH biosynthesis is catalyzed by GSH1, which is regulated at three levels. First, feedback inhibition of GSH1 activity by GSH has often been considered a fundamental control over GSH synthesis. Alleviation of this feedback inhibition under GSH-consuming conditions is possibly an important mechanism driving accelerated rates of GSH synthesis in response to stress [19,35–37]. Under metal stress it is well established that the regulation of GSH biosynthesis undergoes a significant change. Metals increase GSH oxidation and PC production, resulting in a depletion of cellular GSH levels and consequently, the feedback inhibition is released (Figure 1: GSH biosynthesis) [38,39]. Second, *de novo* synthesis of GSH1, but also GSH2, may be enhanced by metal-induced stress [36,39–41]. The 5′-untranslated region of the *GSH1* gene was shown to interact with a redox-sensitive repressor-binding protein that was released upon oxidation [16,37,39,42]. Finally, evidence has been presented that GSH1 activity is regulated via post-translational redox controls. Recently, it has been shown that the plant GSH1 enzyme forms a homodimer linked by two redox-sensitive disulfide bonds (S–S). Although the exact mechanistic details are still subject of discussion, reduction of these bonds (–SH) is suggested to be associated with a conformational change and significant inactivation of the enzyme [20,35,43,44]. This redox regulation probably contributes to the well established upregulation of GSH synthesis in response to oxidative stress conditions like metal toxicity. Exposure of *Arabidopsis* plants to Cd confirms that the distribution of GSH1 is shifted towards the more oxidized and thus active form [45]. However, it remains unclear whether this mode of regulation is the underlying mechanism of feedback inhibition [46].

Two structural features defining GSH are a thiol on cysteine and a γ-glutamyl linkage. Since it is the most abundant intracellular thiol as well as γ-glutamyl compound, important biological functions are evident for GSH. Generally, the physiological significance of GSH in plants may be divided into two categories. First, GSH is an important pool of reduced sulfur and regulates sulfur uptake at the root level. Second, GSH contains one cysteine molecule forming the centre of its biological functions in chelation but also in the antioxidative defense and redox control. The SH-group on cysteine is the link through which diverse defense pathways are combined in GSH that will be further discussed in this review. The distinctive γ-glutamyl linkage prevents GSH from degradation by common proteases. One way of GSH break down, is the transfer of γ-glutamate to other dipeptides, catalyzed by γ-glutamyl transpeptidase [47,48]. In another pathway, glycine is removed from GSH by a carboxypeptidase in vacuoles. The remaining dipeptides are metabolized by dipeptidases [47]. Questions remain about cellular/tissue specificities and activities of degrading enzymes against GSH compared to GSSG or GS-conjugates [46]. Also the influence of metal stress on GSH break down rates is yet to be explored.

## 3. Glutathione and Metal Homeostasis

About one third of all structurally characterized proteins are metalloproteins, having an essential metal in their active centre. However, the same chemical properties that make metal ions indispensable for biological systems are the reason why they can become toxic when present in excess. A tightly controlled metal homeostasis network is needed to adjust fluctuations in metal availability in order to ensure proper distribution of metals and prevent toxic metal accumulation. Upon entering the cell, two mechanisms are present to bind metals: specific chaperones that deliver essential metals directly to their cellular site of action [49,50], and chelators that neutralize and sequester excess free metal ions [51,52]. Although chaperones normally act under control conditions, metal toxicity will also enhance their expression to prevent free damaging metal ions. Excess Cu ions have been shown to induce *COX17* expression [53] and Cd toxicity can increase *CCH* transcripts [54], two genes encoding Cu chaperone proteins. Chelators like GSH and PCs on the other hand, are major contributors to metal detoxification in plants. Therefore, metal toxicity (e.g., Ag, Cd, Cu, Hg) increases their concentrations in order to bind metals and subsequently sequester the ligand-metal complexes [55].

**Figure 2:**
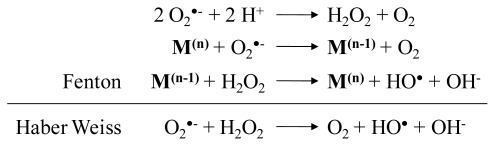
Fenton and Haber Weiss reaction; oxidized transition metal (M^(n)^), reduced transition metal (M^(n−1)^), superoxide (O_2_^•−^), hydrogen peroxide (H_2_O_2_), hydroxyl radicals (HO^•^, OH^−^).

In general, almost all metals strongly bind to thiol groups of cysteine amino acids, making free cysteine an effective chelator of metal ions. However, when bound to a redox-active metal, cysteine is rapidly oxidized and the reduced metal might undergo a Fenton reaction and form highly toxic hydroxyl radicals (HO^•^; Figure 2). Therefore, keeping free cysteine levels low (up to 50 μM) is necessary to protect against these oxidants. This thiol oxidation by transition metals is greatly reduced after blocking the cysteine amino group by conjugation with glutamate and even further with glycine to form GSH. In this way, a cell can contain a low cysteine concentration and high millimolar GSH concentrations without triggering deleterious Fenton reactions [56]. Glutathione protects potentially susceptible cysteine-rich proteins from binding free metal ions and consecutively affecting their function. After forming nontoxic complexes with metals, GSH facilitates their sequestration away from sensitive sites in cells [17,52,57]. Several studies clearly demonstrate the induction of GSH-metal complexes after metal exposure [40,52,55,58,59]. However, not only free metals but also potentially dangerous xenobiotics like herbicides, and metabolites such as anthocyanins, are bound to GSH. The enzyme catalyzing these conjugations is glutathione S-transferase (GST) [47,60]. The activity of GST is shown to be increased in *Arabidopsis* after Cu or Cd treatment in order to stimulate free metal binding [54,61]. In plants, the majority of toxic components are translocated and stored into vacuoles [58,62–64]. A tonoplast multidrug resistance-associated protein (MRP) transporter of the ATP-binding cassette (ABC) type couples ATP hydrolysis to the transport of these GS-conjugates across the vacuolar membrane [36,52].

Phytochelatins (γ-(EC)_n_-glycine) are polymerized forms of 2 to 11 GSH molecules, produced by phytochelatin synthase (PCS), a γ-EC transpeptidase (Figure 1: Chelation). Their multiple thiol-binding sites have increased affinity for metals and render PCs more efficient in chelating several metal ions [36,52]. In leaves of *Arabidopsis* plants exposed to Cd, *PCS1* expression and PC levels (mainly PC2) were strongly induced [39,40]. Also after Cd exposure in liquid culture PC levels were induced in a time and concentration dependent manner, whereas a less pronounced stimulation was found after Cu exposure [39,40]. Metals activate PCS through a metal-specific binding site on the enzyme; removal of the metal ion from PCS inactivates the enzyme again [65,66]. Metal chelation through PCs is an important defense strategy in metal homeostasis as PC-deficient *Arabidopsis* mutants are shown to be hypersensitive to metals like Cd, Hg and As [11,67]. Studies using HPLC data show significant increases in total thiol content after all kinds of metal exposure from different categories *i.e.*, essential *versus* non-essential, redox-active *versus* non-redox-active [68–72]. Although the total thiol concentration is increased, we must distinguish between its four constituents, cysteine, γ-EC, GSH and PCs. Cysteine was increased in *Brassica juncea* seedlings after Cd exposure [72], whereas they remained constant in *Brassica napus* plants exposed to Cd [71] and *Arabidopsis thaliana* plants exposed to Hg [68]. Since free cysteine can be toxic, it is kept at low concentrations in cells due to the activation of its conversion into peptides. The following constituent in GSH biosynthesis, γ-EC, accumulated to a great extent in Cd-exposed plants [71,72], whereas the concentrations of GSH decreased or remained stable upon Cd or Hg exposure [54,68,70]. Together these findings suggest GSH2 to be rate limiting in GSH biosynthesis after metal treatment. Consistent with this hypothesis, data confirm a reduced GSH2 activity after metal treatment [73]. This explains why overexpression of *GSH2* does not affect GSH levels in unstressed plants [38,74] but under metal stress it may alleviate the depletion of GSH and enhance PC synthesis resulting in an increased metal tolerance [38]. Polymerization of GSH into PCs is absent under control conditions but its synthesis is stimulated in a diverse array of plants upon exposure to a wide range of metals [69]. Furthermore, Grill and co-workers (1987) presented a time course of PC induction and GSH consumption after 200 μM Cd(NO_3_)_2_ to a *Rauvolfia serpentina* cell suspension culture, showing a fast response in which GSH is decreased because it is used as a substrate for PC [69].

Long-distance translocation of metals from roots to shoots has been proposed to occur amongst others via the xylem in a PC-dependent manner. These findings suggest that, in addition to the known cellular protection function of PCs, they contribute to maintain a low free metal content in roots. This hypothesis was confirmed by a study of Gong and co-workers in Cd-treated *Arabidopsis* [75]. However, also PCs and Cd were detected in the phloem of Cd-exposed plants, but this retranslocation needs further exploration [59,71]. Phytochelatins are usually associated with detoxification of non-essential metals. However, not only toxic metals, but also the essential ions like Cu and Zn are bound by PCs suggesting a function for PCs in the regulation of their free cellular concentrations [76,77]. A role for Zn metabolism is clearly demonstrated in PC-deficient *Arabidopsis* mutants (*cad1-3* and *cad1-6*) as deficiency in PCs resulted in a pronounced Zn hypersensitivity and a significant reduction in root Zn accumulation [78]. However, contradictory results indicate that metal-induced PC overproduction might deplete GSH to an extent that causes oxidative stress. A study of Semane and co-workers (2007) supports this finding [40]. They observed an increased PC production, combined with a decrease in GSH content and a more oxidized GSSG/GSH ratio after Cd treatment (1 and 10 μM) in *Arabidopsis*. At the same time, several antioxidative enzymes are activated, indicating increased oxidative stress [40].

Phytoextraction studies are looking for safe ways to enhance accumulation of excess metals in plants. An interesting course is the overproduction of chelating and binding proteins such as GSH and PC [79]. Studies confirm that overexpression of enzymes in sulfate assimilation, GSH or PC biosynthesis have led to enhanced metal accumulation and tolerance to different metals [36,80–83]. For example, transgenic *B. juncea* overexpressing *GSH1* and *GSH2* showed enhanced Cd accumulation and was able to significantly reduce Cd and Zn concentrations in metal-contaminated soil due to increased γ-EC, GSH and PCs [38,72,84]. Overexpression of *PCS1* in tobacco plants resulted in enhanced accumulation of Cu, Cd, Pb and Zn in shoots of plants grown on soils polluted with metals [83,85]. However, when only *PCS* was overexpressed, it did not always turn out to be beneficial. In *Arabidopsis* and *B. juncea*, transgenic plants with the highest *PCS* transcript levels paradoxically were hypersensitive to Cd, whereas plants with moderate overexpression were more resistant [23,86]. Additionally, transgenes can behave different in other plant species; *Arabidopsis AtPCS1* showed high resistance to As and hypersensitivity to Cd in *Arabidopsis* [87], but in *B. juncea* this *AtPCS1* improved both As and Cd tolerance [86]. A possible explanation for unexpected or contrasting observations is that forcing the plant to overproduce PCs caused a severe GSH depletion, which disables GSH in fulfilling its other important functions in antioxidative defense and signaling. Also sulfate homeostasis will be disturbed in order to constantly maintain the GSH content. Therefore, metal tolerance is related to the plant’s ability to produce PCs and to prevent associated GSH depletion. In accordance, overexpressing genes involved in sulfate assimilation and GSH biosynthesis generally seemed more successful in enabling plants to overcome metal toxicity [18]. Support was found in metal-hyperaccumulating plants that don’t seem to rely on PCs, but overexpress several antioxidant-related genes and have an enhanced synthesis of GSH to counter the risk of oxidative stress related to high metal uptake [88].

## 4. Glutathione and Antioxidative Defense

Multiple studies have indicated that plants exposed to any of a diverse array of metals elicit oxidative stress, a process in which the cellular redox balance between pro- and antioxidants is disturbed in favour of the former [14,51,89–91]. Uncontrolled increases in the steady-state concentrations of these pro-oxidants lead to free radical-mediated chain reactions that target proteins, lipids, polysaccharides and DNA. It has been suggested that metal-induced oxidative stress in cells is partially responsible for the toxic effects of metals [92]. In order to cope with this oxidative damage, small fluctuations in pro-oxidant concentrations play an important role in signaling processes that regulate cellular responses, resulting in cellular protection and/or acclimation to Cd or excess Cu [89]. Hence, the term “oxidative challenge” is used instead of “oxidative stress” due to the negative connotation of “stress” [12,16,89,93,94].

The primary response of plants to metal stress is the generation of reactive oxygen species (ROS) such as superoxide (O_2_^•−^), hydrogen peroxide (H_2_O_2_) and the hydroxyl radical (HO^•^), the major contributors to oxidative damage [95,96]. Depending on the physico-chemical properties of the metal, ROS are formed by different mechanisms. First, even under non-stress conditions, ROS are unavoidable by-products of cellular respiration. Second, due to their ability to change in oxidation number, free redox-active metals like Cr, Cu and Fe can directly enhance ROS production through Fenton and Haber Weiss reactions (Figure 2) [97]. Furthermore, metals can alter the cellular redox state indirectly via targeting components of the respiratory chain or antioxidant defense system. Finally, metals can activate pro-oxidative enzymes such as NADPH oxidases and lipoxygenases [13,93,98,99]. These last two mechanisms are ways in which also non-redox-active metals (e.g., As, Cd, Co, Hg, Mn, Ni, Pb, Zn) can provoke oxidative stress.

Metal-induced ROS can adversely affect plants at several levels: morphological (e.g., reduced growth, leaf curling), physiological (e.g., photosynthesis, chlorosis, mineral uptake) and biochemical (e.g., membrane leakage, protein inactivation) [51,100–105]. Due to their immobility plants inevitably need to cope with stress conditions, and also the fact that plants both consume and generate oxygen during respiration and photosynthesis is giving them a greater oxidative challenge compared to other eukaryotes. As a result, plant cells respond defensively to oxidative damage by removing ROS and maintaining antioxidant defense compounds at levels that reflect ambient environmental conditions. The antioxidative system contains both enzymatic defenses [e.g., superoxide dismutase (SOD), catalase (CAT), peroxidase, reductase, redoxin] and metabolites [e.g., GSH, ascorbate (AsA)] [13,97,99,106]. Various stress factors, including metals, can disturb the balance between the cellular activities and concentrations of ROS scavengers, leading to cellular damage.

Glutathione is a key player in this antioxidative system, with a significant function in ROS scavenging and as a redox buffer to keep the cellular redox state in balance [36,107,108]. Glutathione exists in reduced (GSH) and oxidized (GSSG) forms. In the reduced state, the thiol group of cysteine is able to donate a reducing electron directly to unstable molecules such as ROS. In donating an electron, GSH itself becomes reactive, but readily reacts with another reactive GSH to form GSSG (Figure 1: Direct H_2_O_2_ detoxification). In a following step, GSH can be regenerated from GSSG by the action of glutathione reductase (GR), at the expense of NADPH. Two genes are annotated to encode GR in plants: cytosolic *GR1* and *GR2,* which is dually targeted to plastids and mitochondria. A key characteristic of the cellular GSH pool is its high reduction state due to GR that is constitutively active and inducible upon oxidative stress (Figure 1: Regeneration). In healthy cells more than 90% of the total GSH pool is in its reduced form [13]. After metal treatment however, GSSG/GSH ratios have been shown to decrease by 65% despite of an increased GR activity [40,90]. This suggests that metal-induced stimulation of GR is insufficient to cope with the massive GSH-consuming effects (direct metal-GSH binding, GSH oxidation, PC synthesis), causing a reduction in free reduced GSH.

In addition to its primary antioxidant capacities, GSH participates in the AsA-GSH cycle that is located in various subcellular compartments (Figure 1: AsA-GSH cycle). This cycle, exclusively existing in plants, is essential for their normal metabolism as well as their defense against oxidative stress. It includes the successive oxidation and reduction of AsA and GSH with a cyclic transfer of reducing equivalents so that the plant-specific ascorbate peroxidase (APx) is able to reduce H_2_O_2_ to H_2_O. The cellular pool of AsA is also maintained in its reduced state by dehydroascorbate (DHA) reductase (DHAR) that uses GSH as an electron donor [13,16,36]. Highly reduced GSH and AsA pools are essential for an optimal function of the AsA-GSH cycle. Transgenic *Nicotiana tabacum* plants overexpressing GR indicate the critical role of GSH, GR and DHAR in maintaining the AsA pool. Although the GSH pool remained reduced in both plants under oxidative stress conditions, nontransgenic plants displayed a more oxidized AsA pool as compared to transgenic plants. It is essential to keep the AsA pool reduced because APx is rapidly inhibited in the absence of AsA. This study supports the essential role of GR in the rate of electron supply to DHA [109,110]. Several studies have demonstrated metal-induced increases in the activities of enzymes involved in this cycle and/or highly oxidized metabolite pools, especially in roots. Therefore, both AsA and GSH act as important redox buffers and their oxidation-reduction ratios reflect the cellular toxicity [40,90,100,111,112]. A study of Cuypers and co-workers (2001) illustrate a fast immediate response of the AsA-GSH cycle to Zn toxicity (50 μM Zn) in *Phaseolus vulgaris*. Already 5 hours after Zn exposure, the roots show elevated DHA/AsA and GSSG/GSH ratios due to a decrease in AsA and an increase in GSSG, respectively. The early GSH oxidation might be the cause of AsA regeneration. However, after 5 hours APx activity was decreased due to a lower total AsA concentration, confirming previous findings [113]. This decrease in antioxidant capacity may induce oxidative stress. After 5 days the total AsA content and APx activity were restored in order to deal with the oxidative challenge but the DHA/AsA ratio remained high, indicating that the oxidative challenge was still active. In conclusion, Zn obviously disturbs the GSH balance and hinders the cell from maintaining the AsA pool in the reduced state [114].

In addition to APx, CAT is another major H_2_O_2_-scavenging enzyme. Compared to APx, it has a low affinity but a high reaction speed for H_2_O_2_. It has the additional advantage that it is not limited by a substrate. These differences in affinity of APx and CAT suggest that APx is responsible for the fine tuning of ROS concentrations for signaling, whereas CAT might remove the excess ROS as was suggested after metal exposure in *Arabidopsis thaliana* [90]. A recent study conducted by Mhamdi and co-workers (2010), provides direct evidence for a role of GR1 in intracellular H_2_O_2_ metabolism. An *Arabidopsis* GR1 knock-out mutant (*gr1*) and a CAT-deficient mutant (*cat2*) are both characterized by increased GSSG concentrations due to a lack of GR1 on one hand and H_2_O_2_ accumulation on the other hand. If the major function of GR is to provide GSH for DHA reduction to AsA, down-regulation of *GR1* should have a similar response as APx deficiency because APx is inhibited in the absence of reduced AsA. When comparing *gr1cat2* [115] and *apx1cat2* [116] double mutants, an ameliorated phenotype was observed in *apx1cat2*. Different effects between GR1 and APX1 deficiency are explained by the fact that H_2_O_2_ is also metabolized through GR-dependent but AsA-independent pathways. Figure 1 displays three different GR-dependent pathways of H_2_O_2_ metabolism: direct GSH oxidation, the GSH-AsA cycle and the redoxin cycle. The first two pathways have been described above. The redoxin cycle, which is important in cellular (H_2_O_2_) and protein (-SH) redox homeostasis, is explained in the next section. Although thioredoxin-(TRx-) and GSH-dependent pathways have overlapping functions in plants [117,118], Mhamdi and co-workers (2010) demonstrated no upregulation of TRx-dependent genes in increased H_2_O_2_ conditions like *cat2* and *gr1cat2*, providing evidence that GR1 plays a specific role in intracellular H_2_O_2_ metabolism. In contrast, GSH-dependent proteins like glutaredoxin (GRx) and GST were transcriptionally upregulated in these mutants [115]. Further work is ongoing to confirm the role of GSH status and GR in H_2_O_2_-trigerred signaling.

## 5. Glutathione Redox Homeostasis and Signaling

Plants constantly face environmental changes as they grow and develop. In order to adapt to their surroundings, plants require both efficient perception and signaling systems. To maintain a reduced state in an otherwise oxidizing environment, plants possess internal redox control systems. Elevated ROS production is a general response in plants exposed to metal stress. Although the detrimental effects of ROS cannot be denied, it is the paradoxical concept that the same reactive radicals participate in signal transduction that has become the subject of current research [16,94]. Identification of specialized ROS-generating oxidases in several organisms further supports this concept. For example, NADPH oxidases are suggested to locally create elevated ROS concentrations after metal treatment [59,119–121]. *Arabidopsis* seedlings exposed to 5 μM Cd or 2 μM Cu during 24 hours showed metal-specific responses in roots at the transcript level. Cadmium toxicity is associated with an upregulation of NADPH oxidase, while excess Cu mainly shows a downregulation of these genes but lipoxygenase genes were induced. These data suggest that metals modulate metal-specific signaling networks in order to regulate adaptive responses [98]. Generation of secondary messengers like H_2_O_2_, that are small and able to diffuse over short distances, is a major mechanism to elicit an intracellular signaling response [122]. Mitogen-activated protein kinases (MAPKs) are specific ROS sensors that link perception of an environmental signal to downstream targets via sequential phosphorylation of proteins, including transcription factors and enzymes. Several data show that MAPK cascades are involved in signaling activated by different metals in order to translate the information into a biological response [123–129]. Jonak and co-workers (2004) demonstrated differential activation of several MAPK pathways by Cd and Cu stress in root cells of alfalfa (*Medicago sativa*). These responses occur within half an hour and activate multiple cellular signaling mechanisms, supporting the statement that the cellular response to metal ions and the following signaling pathways are integrated in a signal transduction process [124].

### 5.1. Redox Control of Protein Function

Although classical signaling pathways depend on macromolecular interactions, mild oxidants such as H_2_O_2_ signal through chemical reactions with functional groups of target proteins, resulting in covalent protein modifications at the atomic level. The thiol residue of cysteine, one of the most common amino acids found in proteins, is very useful for structural and regulatory aspects of cells and at the same time a major site of action for ROS. Oxidation of these sulfhydryl groups in proteins results in disulfide bonds that may be required for proper folding to increase enzyme stability or to maintain its activity. Continuous oxidation and reduction of these S–S is possible due to fast and reversible electron transfer between the active site cysteines of thiol-redox enzymes and the cysteines in the target protein [130]. Two major thiol-redox enzymes are the NADPH-dependent GRx and TRx (Figure 1: Redoxin cycle). Both systems complement the GSH system in determining protein thiol/disulfide status, a primary factor in redox signaling. In addition, they protect thiol-containing proteins from irreversible oxidation during severe stress conditions (Figure 1: Redox control by protein thiols) [131]. Although metal-induced TRx and GRx activation has not been studied intensively in plants, there are indications that GRx is activated by As [132,133]. Thioredoxins use two cysteine residues in their active site to reduce protein disulfides. Their active site is reduced in its turn by a ferredoxin- or NADPH dependent thioredoxin reductase (FTR and NTR, respectively). Glutaredoxin on the contrary, can also reduce thiols via reversible glutathionylation of the proteins (*i.e.,* the formation of a disulfide bond between GSH and specific cysteine residues) and can be reduced by GSH and NADPH-dependent GR [134–137]. Also peroxiredoxin (PRx), which catalyzes the reduction of H_2_O_2_, is recycled by thiols from GSH, GRx and TRx [138]. Only a few studies have investigated the effect of metal toxicity on PRx and show increased transcript levels after treatment with Cd or Cu [139–141]. Additionally, recent findings using NTR-knockouts and GSH-deficient mutants identified an alternative reduction of TRx by the GSH-GRx pathway and an NADPH-dependent TRx system as a backup system for GR in *Arabidopsis* [117,118,131,142]. In this regard, GSH is essential for the regeneration of the redoxin pool in plants [15]. These dithiol-disulfide transitions, including glutathionylation, are the major mechanism in redox control of protein function [130,138,143].

### 5.2. Cellular Redox Control

Antioxidant redox buffering homeostatically regulates ROS signaling via dithiol-disulfide transitions. In order to maintain the total cellular redox balance, ROS and antioxidants are strongly connected at the level of transcription and translation. Depending on the environmental condition encountered by plants, *i.e.*, biotic (bacteria, fungi…) or abiotic (metals, heat…) stress, they may alter the balance between ROS production and removal to enhance or suppress the cellular level of ROS respectively [16].

Cells require a reducing environment that provides the electrochemical gradient needed for electron flow in order to survive. To keep the internal medium in a reduced state, biological evolution created a complex redox buffering system. Many redox couples in a cell (e.g., oxidized/reduced NAD(P), TRx, GRx) work together to provide electron transfer in order to maintain the cellular redox state. The GSSG/GSH couple is the most abundant in cells and is therefore considered the principal cellular redox buffer. However, the GSH-, TRx- and GRx-systems use NADPH as a source of reducing equivalents, demonstrating an interesting thermodynamical connection between these systems (Figure 1: Regeneration). The biological status of a cell is closely linked with its redox environment. The redox state of GSSG/GSH serves as an important indicator of the redox environment, defined by its redox potential (E_GSSG/2GSH_ = −269.55 log([GSH]^2^/[GSSG]) mV at 25 °C and pH 7.0) and its reducing capacity (total GSH concentration). The redox potential of the cellular environment (E_hc_) is a measure of the tendency of the GSH redox system to acquire electrons and thereby being reduced. The higher the potential, the more oxidized the GSH redox pool is and therefore the greater its affinity for electrons [108,144]. The redox environment of a cell changes throughout its life cycle; recent findings suggest that metabolic oxidation regulates the cell cycle and embryonic stem cell differentiation in animals [145,146]. Since very similar patterns of GSH recruitment into the nucleus have been observed in plant and animal cells, these mechanisms are possibly the same in all eukaryotes [147–149]. At the G1 phase of the cell cycle, GSH is recruited into the nucleus in both plant and animal cells. Cytosolic depletion of GSH causes a readjustment of the intracellular redox environment and oxidative signaling [149]. Immediately, GSH significantly accumulates throughout the cell [150], suggesting activation of GSH biosynthesis due to GSH depletion coupled to stromal oxidation. Foyer and Noctor (2011) suggest that posttranslational GSH1 activation and the observed enhanced *GSH2* expression [149], lead to the increased GSH production and the larger total GSH pool required for redistribution between the daughter cells following mitosis [17,148].

Schäfer and co-workers (2001) developed a theoretical scale of physiological states ranging from cell division over differentiation to cell death, in which E_GSSG/2GSH_ is regarded as a trigger to activate cellular switches between these states. The start of plant development is characterized by proliferating cells with high GSH levels and the most negative redox environment (E_hc_ < −240 mV) [108,144]. Metal-induced oxidative stress can cause plants to display slow-growth phenotypes resulting from an increased oxidative load. Evidence was found in CAT-deficient *Arabidopsis* mutants with reduced growth that is linked to GSSG accumulation instead of an increased H_2_O_2_ production [151]. This GSSG accumulation suggests that these mutants are not capable of maintaining the GSH status at sufficient values to allow the dividing cells to progress rapidly out of the G1 phase [17]. Glutathione abundance in proliferating cells plays a critical role in development via regulating auxin transport and signaling [15]. Studies on metal-induced changes to the cell cycle in plants explain their reduced growth. After treatment with Pb (2.5 mg/L during 30–72 hours), *Allium cepa* L. showed a prolonged cell cycle by 55 to 216% depending on the cell division phase of the cells [152].

More positive redox potentials (E_hc_ from −240 to −200 mV) slow down proliferation and activate nano-switches for differentiation until a maximum is reached and nearly all cells are moved from proliferation to differentiation [108,144]. The control of the intracellular redistribution of antioxidants, especially GSH, has been suggested to act as a signal in the regulation of the cell cycle [17]. However, not many studies have been performed in this area on plants exposed to metals. Cells that are not terminally differentiated could proliferate again when an appropriate signal and associated, more negative, redox environment are apparent. Additional increases in E_hc_ can suppress differentiation and when the redox environment cannot be maintained due to stress or damage, death signals are activated and apoptosis is initiated (E_hc_ from −200 to −170 mV). When cells are not capable of activating or responding to these switches, severe oxidative stress will greatly increase the redox potential and necrosis will occur (E_hc_ > −170 mV) [108]. From this point of view, the redox environment is a useful tool to determine biological vitality [153]. A study shows that in general, plant stress becomes lethal when E_GSSG/2GSH_ exceeds −160 mV due to a signaling cascade initiating apoptosis. Although E_GSSG/2GSH_ is proposed as a universal marker of plant viability and to predict whether seeds may live or die, only few studies are published using this marker [154,155]. Several studies demonstrate that the cellular redox state is an important characteristic of metal phytotoxicity [39,90,156]. Treatment of *Arabidopsis* with 1 μM Cd during 1 week showed increased GSSG and reduced GSH concentrations, resulting in an elevated GSSG/GSH ratio. These plants seemed able to cope with the metal stress and adopt a new metabolic equilibrium. Plants exposed to 10 μM Cd however, were not capable of maintaining the redox homeostasis and suffered from metal-induced oxidative stress [40]. For both high and low metal concentrations it is interesting to measure E_GSSG/2GSH_ after metal treatment in order to determine cell viability in future studies.

The assessment of the redox environment is straightforward in homogenous fluids. In cells however, compartmentalization of GSH and GSSG influences the local GSSG/GSH ratio. For example, the endoplasmatic reticulum has a more oxidizing environment (E_GSSG/2GSH_ = −180 mV) with respect to the cytosol (E_GSSG/2GSH_ = −232 mV) in order to support and ensure proper protein folding and formation of necessary disulfide bridges [108,157]. In addition, depending on the total GSH concentration and pH in a cell or organelle, the size of an oxidative event associated with nano-switches will vary. Therefore, findings often seem confusing and contradicting because a certain treatment might change the biological status of one cell while no response occurs in another. However, one must bear in mind that not all oxidative stimuli create a more oxidized biological state, mild oxidants like H_2_O_2_ are normal regulators of cellular homeostasis until the capacity to detoxify them is exceeded [108,144]. Although metal-induced subcellular redistribution of GSH is still in its infancy, the following paragraph summarizes the latest findings.

### 5.3. Glutathione Compartmentalization

In order to fully understand the function of GSH in signaling networks, the subcellular compartmentalization of this key redox player should be further investigated. Immunolocalization studies with specific GSH-fluorescent labeling revealed that under control conditions GSH is present in all cellular compartments of root and leaf cells, with the exception of the apoplast [158,159]. At the macroscopic level, GSH is also detected in phloem vessels and vascular parenchyma cells, indicating GSH loading into phloem cells and transport to other plant parts via sieve tubes. Since GSH was not detected in cell walls or intercellular spaces, the loading is suggested to take place through plasmodesmata [159]. An oligopeptide transporter (OPT) has been introduced as transporter of GSH, GSSG and even GS-conjugates across the plasma membrane in *B. juncea*, rice and *A. thaliana* [46,160,161]. Consistent with its proposed role in long-distance transport, *OPT6* is highly expressed in the vasculature [161]. Redundant genes are proposed for OPT6 because knockout mutants display no altered phenotype [162]. As mentioned before, combination of Cd and GSH/PCs in xylem sap of Cd exposed plants has been observed, indicating long-distance transport of GSH under metal stress [59,71,75].

Glutathione biosynthesis is restricted to the cytosol and plastids, so the newly synthesized GSH must be distributed to other subcellular compartments. Transport of both γ-EC and GSH across the plastid envelope by the chloroquinone-like transporter (CLT) family confirms the finding that GSH1 is exclusively located in the plastid and the major location of GSH2 is the cytosol [26,46,163]. Recently, the highest GSH levels were detected in mitochondria, followed by the nucleus [158,159]. Recruitment of GSH into these compartments suggests the presence of proteins that can increase the permeability of pores for GSH sequestration in the nucleus and mitochondria as is observed for animal cells [46,164]. Findings of Zechmann and co-workers (2010) that GSH concentrations are the highest in mitochondria, and that even in situations of permanent GSH deficiency, these levels are maintained at the expense of other subcellular GSH pools is interesting for future research. This effect was observed in both plant and mammalian cells using the GSH-deficient *Arabidopsis* mutant *pad2-1* (*in vivo*) or the GSH1 inhibitor BSO (*in vitro*) [158,165,166]. Since mitochondria cannot synthesize GSH themselves [26], this finding demonstrates highly competitive mitochondrial GSH-uptake systems. In *Arabidopsis* it is known that mitochondria play a central role in the cellular carbon and nitrogen metabolism. Changes in mitochondrial electron transport and/or mitochondrial ROS production can influence all other organelles [167,168]. Mitochondria are highly sensitive to redox fluctuations due to Cd toxicity as shown after exposure of *Arabidopsis* cell cultures to 5 μM Cd. In this experiment Schwarzländer and co-workers (2009) demonstrated a redox perturbation in the mitochondria after Cd exposure with important effects on redox signaling. Two possible explanations were proposed. There is either a reduced capacity of the mitochondria to buffer oxidation, or there is a persistent ROS production after treatment due to oxidative damage to the electron transport chain [169]. Additionally, mitochondria are required to process excess reductants to form a proton gradient across the membrane for respiration [170]. In order to prevent massive oxidative damage, mitochondrial O_2_^•−^-neutralizing MnSOD is accompanied by H_2_O_2_-scavenging components. The AsA-GSH cycle plays a major role in mitochondria; both enzymes and metabolites of this cycle have been shown to be affected after metal stress [18,90]. Together, this suggests an essential role for mitochondria in both perception and signaling after metal-induced oxidative challenges in plants [167–169]. Mitochondria acting as a sink for GSH may thus play an important role in signaling and H_2_O_2_-detoxification [165,166] and therefore be an important survival strategy to prevent cytochrome *c*-induced cell death [171,172].

To investigate the influence of Cd on the subcellular GSH compartmentalization, Kolb and his co-workers (2010) published a study in which *Cucurbita pepo* L. was exposed to 50 μM Cd during 48 hours. Under control conditions, highest GSH-directed immunogold-labelling density was detected in mitochondria, followed by the nuclei as previously described [158,159,166]. Consistent with previous experiments [40], all organelles show a strong decrease in GSH content after Cd treatment, reflecting the importance of GSH in Cd-detoxification. The absence of labelling in vacuoles demonstrates that GSH-metal complexes, which are not recognized by the present immunohistochemical approach, are sequestered to this compartment after complexation in the cytosol. The well established decrease in free GSH due to Cd toxicity is thus a consequence of, among others, PC synthesis and metal complexation [173]. In this study, a differential Cd-induced compartmentalization of GSH was found between mesophyll and glandular trichome cells. All organelles from both cell types show a GSH reduction of 30 to 40%, except for the GSH content in nuclei and cytosol of trichome cells, which was decreased between 70 and 76%. These results indicate a possibly crucial role in Cd detoxification for GSH located in these compartments in trichome cells [173]. Other studies support that glandular trichomes can accumulate and even excrete large amounts of metals and might be considered the major compartment of Cd accumulation in leaves [174,175]. The cytosol is important for Cd detoxification in these cells since the first contact with metals after uptake into cells occurs here and it is the major compartment for PC synthesis [173]. A different study using a CAT2-knockout *Arabidopsis* mutant with increased H_2_O_2_ levels provides evidence that GSSG accumulation in vacuoles and chloroplasts influences the subcellular distribution of GSH as a response to oxidative stress. This GSSG compartmentalization may play a role in helping to maintain a reduced cytosolic GSH redox status, implying that plant cells are configured to limit large changes in the cytosolic (and possibly nuclear) GSH redox potentials [46,151,176,177]. Additionally, GSSG sequestration may partially explain why plants can tolerate GSSG increases without inducing cell death [178,179]. Although GSH concentrations in the vacuoles of unstressed plants have long been considered to be low or negligible [158], accumulation of GSSG in this compartment could be a physiologically important part of oxidative stress responses [176]. This vacuolar import suggests MRP transporter activity will be increased in response to stress-induced increases in cytosolic GSSG accumulation and function to reduce such increases. The expression level of several MRP proteins (MRP3, MRP6 and MRP7) is significantly upregulated in *Arabidopsis* roots after 5 μM Cd exposure. Consistently, two knockout *Arabidopsis* mutants *mrp6*.1 and *mrp6*.2 display more disturbed leaf development when treated with Cd in comparison with the wildtype [180]. However, the role of GSSG accumulation in the vacuoles and plastids remains to be investigated. Moreover, it remains unclear why equal decreases occur in nuclei [173]. The exact role of GSH in the nucleus on plant cells is not fully understood. Nevertheless, high ROS and Cd accumulation seem to be unlikely as Cd accumulates mainly in cytosol and vacuoles [59]. The authors suggest a Cd-induced relocation of GSH from nuclei to the cytosol for complexation. This statement is supported by the large decrease in GSH of more than 50% in both nuclei and cytosol of Cd-exposed trichome cells [59,158,166,173]. A missing link is the detection or labelling of GSSG and GS-conjugates that can be used in future and more detailed experiments in plants under metal stress.

### 5.4. Glutathione Signaling

The antioxidant status appears to set the threshold for general plant defense responses. Any stimulus that changes the cellular redox balance may induce the same set of defense-related genes as ROS. Glutathione status in particular has been proposed to be important in relaying oxidative signals originating from ROS. Several studies suggest that changes in GSH status and enhanced ROS pools are equally important in redox signaling [181–183]. Depending on the capacity of the cellular GSH buffer, either a signal is perceived and relayed, resulting in signaling cascades that elicit downstream responses, or signals may be attenuated and even stop [144]. However, the exact mechanism of when and how this system relays signals is still under investigation.

Currently, at least two mechanisms have been described by which GSSG concentration or the GSH status can modulate the activity of signaling proteins independently of ROS. Both are posttranslational modifications that modify cysteine residues in catalytic sites or compete with other thiol modifications in order to regulate enzyme activity. First, GSH can change the redox state of thiol groups of proteins that act as redox cofactors. These so-called thiol switches or protein thiol/disulfide exchanges alter either the activity or the redox state of regulatory proteins. Although, elevated protein oxidation is usually associated with oxidative stress, recent findings reveal a fundamental role for this modification in cell signaling. This reversible process is called redox regulation, analogous to the well described phosphoregulation [184]. In plants, TRx is known to regulate several enzymes in this way, however GRx also couples the GSH redox potential to these changes in thiol/disulfide state [19]. Although plants contain a large GRx family, much remains to be discovered regarding their functions under normal and environmental stress conditions like metal stress [185]. Microbial thiol switches have been intensively reviewed [186], but for plants only a few studies are conducted. A first study, as mentioned before, demonstrates that GSH1 from *Nicotiana tabacum* forms an active homodimer under oxidizing conditions [43]. Another study hypothesizes that an increased GSSG/GSH state serves as secondary messenger connecting the salicylic acid signal induced by a plant systemic acquired resistance with the activity of the nonexpressor of pathogenesis-related protein 1 (NPR1). The activity of the latter is increased after reduction of this oligomer into a monomeric form, possibly accomplished by the oxidized GSH pool [183].

A second mechanism is glutathionylation of regulatory proteins with direct conjugation of GSH to target cysteine residues, another function of GRx [187]. Increased GSSG or ROS-induced generation of protein thiol radicals both trigger protein glutathionylation. It has been suggested that this process is an important mechanism in sensing and signaling redox disturbances such as increased ROS production [138,143]. Photorespiration causes *Arabidopsis* plants deficient in H_2_O_2_-neutralizing CAT (*cat2*) to display greatly enhanced GSSG levels that can accumulate up to 90% of the detectable GSH pool, illustrating a close link between H_2_O_2_ production and changes in GSH status [29,37,176,179]. Semane and co-workers (2007) demonstrated enhanced GSSG/GSH levels and GSSG accumulation in *A. thaliana* after 1 week Cd exposure (1 or 10 μM) [40], resembling the effects of *cat2* suggesting analogous responses. Several hypotheses are proposed that explain this GSSG accumulation. It is suggested that GSSG accumulates because the capacity of GR is insufficient to keep up with H_2_O_2_-detoxifying enzymes, either because the latter have higher capacities or due to redundancy (APx, CAT, PRx). Others indicate that decreases in NADPH/NADP could influence the redox state of the GSH pool. In conclusion, this limitation of GR activity might be important to allow rapid and sensitive changes in GSSG/GSH in order to fulfill signaling by GSH [19,188]. Glutathionylation and its significance *in vivo* remains to be revealed in future research work. An interesting example of glutathionylation has been reported by Michelet and co-workers (2005). A specific type of TRx (f-type) has been shown to be glutathionylated on a cysteine residue away from its active site, resulting in a decreased activity towards specific target proteins including NADP-glyceraldehyde-3-phosphate dehydrogenase (GAPDH) [189]. Suggestions have been made that GRx is also involved in deglutathionylation [190]. Whether these are important mechanisms of GSH signaling under metal stress needs yet to be revealed.

The first direct evidence that the GSH metabolism and the expression of other components of antioxidative defenses are tightly linked was provided in the *Arabidopsis* mutant regulator of APx2 (*rax1-1*). The *rax1-1* mutant is characterized by an enhanced *APX2* gene expression due to a lesion in the gene encoding GSH1 concomitantly with a 50% reduced GSH content. In addition, elevated gene expression of *APX1* and *DHAR1* has also been reported under control conditions in this GSH-deficient *rax1-1* [181]. Cuypers and co-workers (2011) show that Cd (5 and 10 μM) and Cu (2 and 5 μM) stress elicit analogous increased effects on *APX2* expression levels in *Arabidopsis*. Additionally, in the leaves there was an increased *APX1* expression [90]. Experiments to further elucidate the effect of GSH deficiency after metal treatment are currently conducted in our laboratory in order to gain a better knowledge on GSH and its multiple roles in plants under metal stress.

## 6. Conclusions

**Figure 3:**
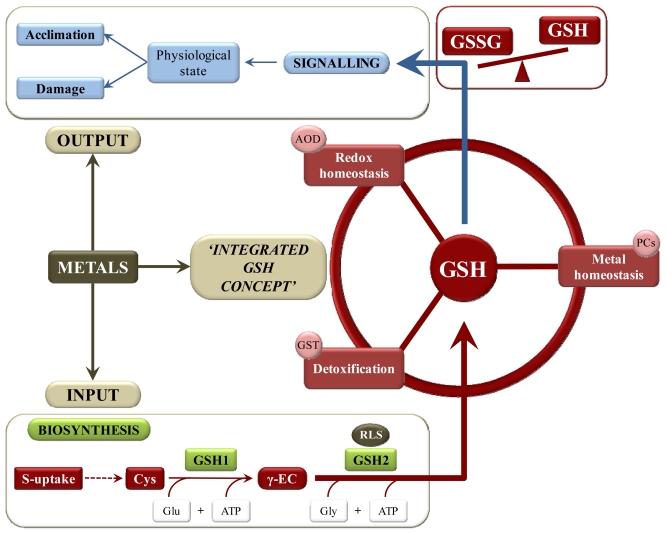
Schematic overview of the three pillars interconnected through GSH and stimulated under metal stress: metal homeostasis, redox homeostasis and detoxification. The demands imposed by this “integrated GSH concept” under metal stress affect the input into the GSH pool and the output into signal transduction pathways. Metals stimulate the input via an increased GSH biosynthesis including sulfate (S) uptake and assimilation into cysteine. Additionally, metal stress shifts the rate-limiting step (RLS) from GSH biosynthesis from GSH1 to GSH2. There are several indications that the GSH state, including both total GSH content and GSSG/GSH ratio, is involved in these signaling pathways. However, future research is necessary to confirm the role of GSH state in signaling and downstream responses under metal stress. Abbreviations: antioxidative defense (AOD), cysteine (Cys), glutathione S-transferase (GST), glutathione synthetase (GSH2), γ-glutamate (Glu), γ-glutamylcysteine (γ-EC), glycine (Gly), γ-glutamylcysteine synthetase (GSH1) and phytochelatins (PC).

Plants are constantly exposed to environmental challenges; tuning of adaptive responses requires an information cascade that starts with efficient signal perception. For several years, GSH has been considered a central molecule in cellular metabolism and signaling. Many publications are available that demonstrate the complex and integrated regulation of GSH status after environmental triggers, including metal toxicity. This review focuses on three pillars that are interconnected through GSH as a core component in the plant’s defense system under metal stress: metal homeostasis, redox homeostasis and detoxification (Figure 3). Not only metals but also other toxic components like xenobiotics are chelated and detoxified by GSH and its derivatives. The cellular redox homeostasis comprises the GSH-regulated antioxidative defense.

The demands imposed by this “integrated GSH concept” under metal stress affect the input into the GSH pool and the output into signal transduction pathways. Glutathione is directed at the biosynthesis level (total GSH content) and by its cellular redox state (GSSG/GSH), both affected by metals. Upstream signals are sensed by GSH and, depending on the GSH buffering capacity, transmitted into a downstream response. Both dose and duration of the disturbance in the GSH redox state determine these downstream pathways and the outcome of the signaling pathway: damage *versus* acclimation. Glutathione’s strategic position between metal scavengers (GSH and PC), detoxification mechanisms (GST) and cellular reductants (AsA-GSH cycle and antioxidative enzymes) makes the GSH redox system (GSSG/GSH) perfectly integrated for signaling functions.

Metal toxicity has been demonstrated to affect GSH at all levels: sulfate uptake and assimilation, biosynthesis of cysteine, GSH and PC and an altered GSSG/GSH balance. Studies using either transformants overexpressing GSH biosynthesis genes or GSH-deficient mutants revealed significant breakthroughs in our understanding of GSH in plant’s metabolism. In assessing responses to metal stress, future research should exploit these systems to elucidate the output of the “integrated GSH concept” *i.e.*, GSH signaling and downstream responses under metal stress.
